# 1st report of unexpected true left-sided gallbladder treated with robotic approach

**DOI:** 10.1016/j.ijscr.2019.04.026

**Published:** 2019-04-16

**Authors:** Antonio Gangemi, Roberto Bustos, Pier Cristoforo Giulianotti

**Affiliations:** Division of General, Minimally Invasive and Robotic Surgery, Department of Surgery, University of Illinois at Chicago, 840 S. Wood Street, Suite 435E (MC 958), Chicago, IL 60612, USA

**Keywords:** Robotic, Sinistroposition, Gallbladder, Cholecystitis, Left-sided, Cholecystectomy

## Abstract

**Introduction:**

True left-sided gallbladder (T-LSG) occur when the gallbladder is positioned to the left of the ligamentum teres and falciform ligament and under the surface of the left liver lobe.

**Presentation of case:**

Patient is 29-year-old caucasian male, presenting with 9-month history of epigastric right upper quadrant (RUQ) colic pain. RUQ Ultrasound reported cholelithiasis, gallbladder wall thickening, and no intrahepatic biliary dilation.

**Discussion:**

Robotic cholecystectomy was the chosen approach. When visceral surface of the liver was exposed, anomalous location of the gallbladder was noted, left to the round ligament. A cystic duct with a “hairpin” configuration and a very cephalad cystic artery were identified. Cholecystectomy was performed safely and uneventfully.

**Conclusion:**

No change of port setting was required with the robotic approach. The ICG-aided cholangiography improved surgeon’s ability to recognize the concomitant vascular and biliary anomalies. However, no definitive conclusion can be drown until further experience and volume are achieved.

## Highlights

• For left-sided gallbladder, no change of port setting is needed using the robot.
• ICG helps to recognize associated vascular and biliary anomalies.
• Anatomical variations assessment is crucial to avoid biliary or vascular injuries.

## 1 Background

True left-sided gallbladder (T-LSG) occur when it is positioned to the left of the ligamentum teres and falciform ligament and is located under the surface of the left liver lobe segments III (or II); situs viscerum inversus is not present in the most common type of LSG [1]. Incidence of this rare anomaly ranges from 0.04 to 1.1% [1,2]. The sinistroposition of the gallbladder is usually discovered intraoperatively producing unexpected challenges during surgery. Despite the fast growing of robotic surgery worldwide, laparoscopic cholecystectomy remains the gold standard for benign gallbladder disease [3]. The intraoperative diagnosis of a T-LSG in the laparoscopic setting, usually requires a change of port setting and it is associated with increased risk of bile duct injuries [4]. Herein, we report the first case of T-LSG successfully treated with the standard robotic approach used for routine right-sided gallbladders. This work has been reported in line with the SCARE criteria [5].

## 2 Methods

Patient is 29-year-old caucasian male, presenting with 9 month history of epigastric right upper quadrant (RUQ) colic pain occasionally radiating to his back, who was admitted to gastroenterology service for worsening abdominal pain. The patient denied any other past medical or surgical history. Laboratory results showed elevated WBC (16.700 cells/mm³), liver function test within normal limits. RUQ Ultrasound reported cholelithiasis, gallbladder wall thickening, and no intrahepatic biliary dilation.

Surgical consultation was requested, and indication for urgent cholecystectomy was established. The robotic approach was chosen based on multiple reasons: – our prior published work that indicates a significant decrease of open conversion and a lower rate of biliary injuries with robotic vs. laparoscopic approach to acute gallbladder disease [10] – our status of high volume center of excellence in robotic surgery; – the academic mission of our minimally invasive surgery center that provides most of the robotic training for general surgery residents at the main university hospital where this case was treated.

## 3 Results

**Fig. 1:**
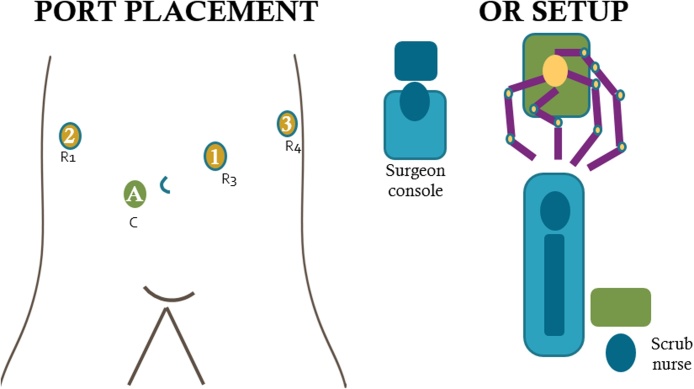
Port placement and OR setup.

**Fig. 2:**
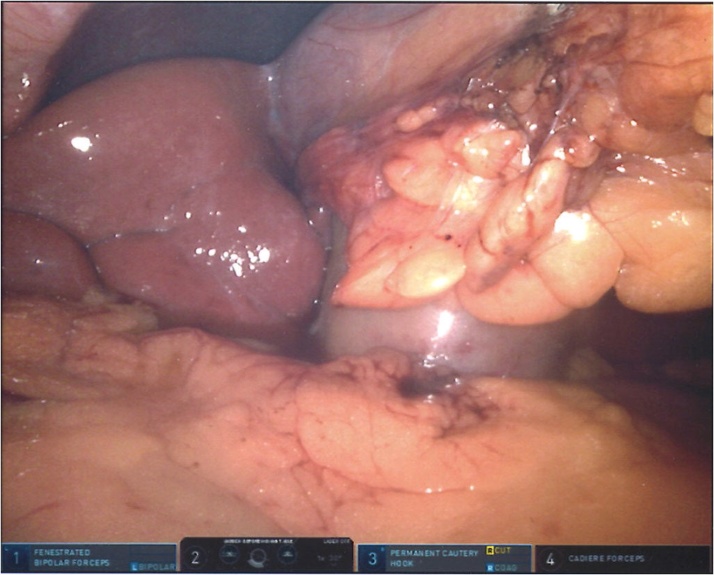
Initial visualization after port placement.

**Fig. 3:**
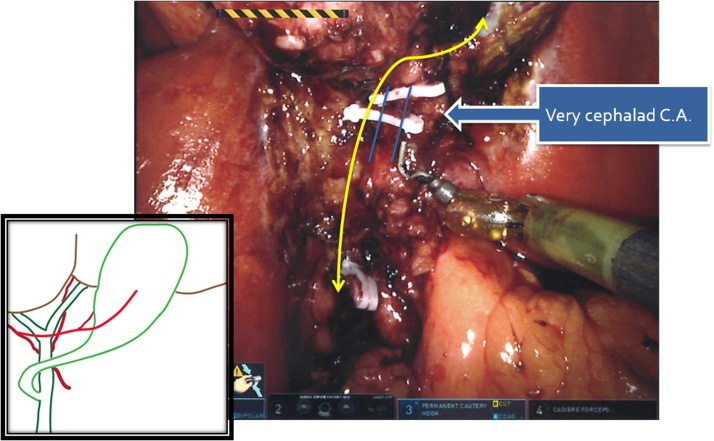
Intraoperative view after CD and CA transection. CD with Hairpin configuration.

**Fig. 4:**
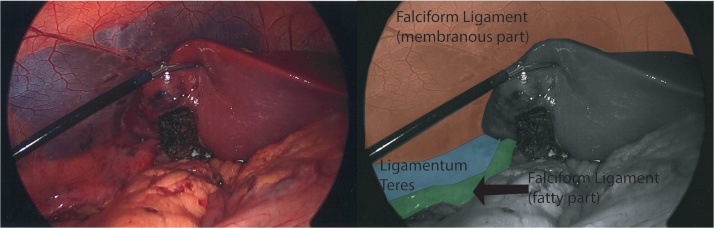
Post Cholecystectomy view.

Da Vinci Xi® Surgical System (Intuitive Surgical, Sunnyvale, CA) was used. After pneumoperitoneum was achieved by placement of Veress needle at Palmer’s point, initial laparoscopy was performed. As gallbladder was not visualized in its usual location coming from the visceral surface of the right liver, we interpreted that the right lobe was covering the gallbladder fundus. Robotic trocars were placed sequentially in the standard fashion over: RUQ, right paraumbilical area, left upper quadrant, and left flank (Fig. 1). When visceral surface of the liver was exposed, anomalous location of the gallbladder was noted (Fig. 2), left to the round ligament, with a more caudal position and the fundus located far from the liver edge. Indocyanine Green (ICG) cholangiography was used to aid with the identification of the biliary anatomy. A cystic duct with a “hairpin” configuration (Fig. 3) and a very cephalad cystic artery (CA) were identified. Cholecystectomy was performed starting with lateral to medial dissection of the hilum. CA and duct were transected in between Hem-o-locks, and the gallbladder safely dissected from its liver bed. No change of standard port setting was required to successfully and safely complete the procedure (Fig. 4).

## 4 Discussion

When sinistroposition of the gallbladder is identified, also associated anomalies involving biliary ducts and vascular supply has to be expected [1].

Regarding biliary duct anomalies, the cystic duct in T-LSG usually joins the CBD on the right side in a right-to-left manner, this is described as “hairpin” configuration [6] (as it was encountered in this case). Less commonly, it may join the CBD or left hepatic duct form the left side [1]. Possible variants involving the CBD also may be associated, such as duplication, hypoplasia and infraportal position [7,8].

When biliary anatomy needs to be assessed, intraoperative cholangiography remains the gold standard, but it has limitations (i.e., difficulty interpreting images, longer operative times, radiation exposure) [9]. ICG cholangiography can be done in real-time, it’s integrated to the robotic console, doesn’t require the use of fluoroscopy or X-rays virtually eliminating the radiation exposure to the patient and the operating room team. Our previous experience reported that 2.07% of patients who underwent robotic cholecystectomy had biliary anomalies identified through ICG cholangiography [10].

Described vascular anomalies of the CA associated to T-LSG are: narrow and long CA; lack of CA; CA crossing in front of the CBD or anterior to the cystic duct; and CA posterior, lateral and very cephalad to CBD [11]. In this case, there were no concerns about the pathway and vascular nature of the pulsatile and hilar structure we identified as cystic artery, but if necessary, intraoperative fluorescent angiography can and has been implemented elsewhere [12].

**Fig. 5:**
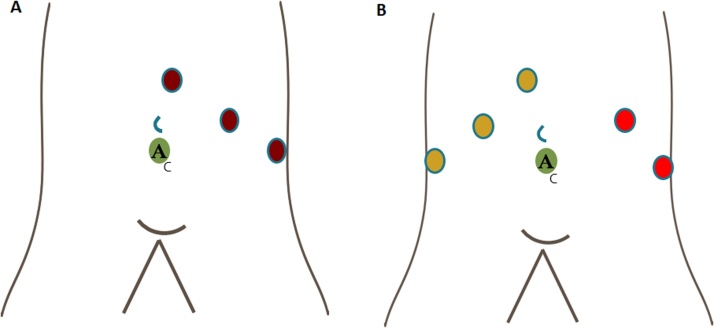
Port setting modification. A) Mirror image setup; B) Additional ports placement.

When T-LSG is identified using laparoscopic approach, most reports suggest to change the port setting. Some authors suggest a mirror image setup, and others decided to place additional ports [4] (Fig. 5). Although conversion has to be considered according to the surgeon’s experience, it is also described to refer patient to a tertiary center after the insertion of cholecystectomy tube, especially when high risk of biliary/vascular injury is suspected [13].

Our first reported case of robotic cholecystectomy with T-LSG seem to support the notion that no change of port setting is required with the robotic approach and that the ICG-aided cholangiography may improve surgeon’s ability to recognize the concomitant vascular and biliary anomalies that are almost invariably associated with T-LSG. These findings are consistent with recent literature data suggesting a benefit of the robotic approach in treating benign gallbladder disease in the acute setting [10,14,15].

Additional technical advantages associated with the robotic platform are: – greater vision of the field featuring 3D images that are filtered and cleaned up by a computer prior to being presented to the operator at the console – the availability of the Endowrist^®^ that reproduces all the degrees of freedom of the human wrist – the routine use of the 3rd robotic arm that greatly facilitates exposure of the tissues.

However, no definitive conclusions on the (potentially) advantageous implementation of robotic approach for the treatment of T-LSG can be drown until further experience and volume are achieved.

## Conflicts of interest

Pier Cristoforo Giulianotti has a consultant agreement with Covidien Medtronic and Ethicon Endosurgery, and he also has an institutional agreement (University of Illinois at Chicago) for training with Intuitive. Antonio Gangemi and Roberto Bustos have no conflicts of interest or financial ties to disclose.

## Funding

This research was not conducted with any specific grants from funding agencies in the public, commercial, or not-for-profit sectors.

## Ethical approval

In its current form this case report does not require ethical approval.

## Consent

Written informed consent was obtained from the patient for publication of this case report and accompanying images. A copy of the written consent is available for review by the Editor-in-Chief of this journal on request.

## Author contribution

Antonio Gangemi: study design, writing, revision.

Roberto Bustos: data collection, writing.

Pier C. Giulianotti: data interpretation, writing, final revision.

## Registration of research studies

It is a case report which doesn’t necessitate a registry.

## Guarantor

Antonio Gangemi.

Roberto Bustos.

## Provenance and peer review

Not commissioned, externally peer-reviewed.
